# Vicarious traumatization in healthcare providers in response to COVID-19 pandemic in Kelantan, Malaysia

**DOI:** 10.1371/journal.pone.0252603

**Published:** 2021-06-04

**Authors:** Mohd Noor Norhayati, Ruhana Che Yusof, Mohd Yacob Azman

**Affiliations:** 1 Department of Family Medicine, School of Medical Sciences, Universiti Sains Malaysia, Kubang Kerian, Kelantan, Malaysia; 2 Raja Perempuan Zainab II Hospital, Kota Bharu, Kelantan, Malaysia; Waikato Institute of Technology, NEW ZEALAND

## Abstract

**Background:**

In the fight against the COVID-19 pandemic, frontline healthcare providers who are engaged in the direct diagnosis, treatment, and care of patients face a high risk of infection yet receive inadequate protection from contamination and minimal support to cope with overwork, frustration, and exhaustion. These problems have created significant psychological and mental health concerns for frontline healthcare providers. This study aimed to compare the levels of vicarious traumatization between frontline and non-frontline healthcare providers in response to the COVID-19 pandemic.

**Methodology:**

All the subjects who met the inclusion criteria were recruited for this comparative cross-sectional study, which was conducted from May to July 2020 in two hospitals in Kelantan, Malaysia. A self-administered questionnaire, namely, the Malay-version Vicarious Traumatization Questionnaire and the Medical Outcome Study Social Support Survey were utilized. A descriptive analysis, independent *t*-test, and analysis of covariance were performed using SPSS Statistics version 26.

**Results:**

A total of 160 frontline and 146 non-frontline healthcare providers were recruited. Vicarious traumatization was significantly higher among the non-frontline healthcare providers (estimated marginal mean [95% CI]: 79.7 [75.12, 84.30]) compared to the frontline healthcare providers (estimated marginal mean [95% CI]: 74.3 [68.26, 80.37]) after adjusting for sex, duration of employment, and social support.

**Conclusion:**

The level of vicarious traumatization was higher among non-frontline compared to frontline healthcare providers. However, the level of severity may differ from person to person, depending on how they handle their physical, psychological, and mental health. Hence, support from various resources, such as colleagues, family, the general public, and the government, may play an essential role in the mental health of healthcare providers.

## Introduction

On March 11, 2020, the World Health Organization confirmed that coronavirus disease 2019 (COVID-19) had spread across the world and declared (COVID-19) a global pandemic [1, 2]. The first case of COVID-19 was reported in Wuhan, China, around mid-November to December 2019 [3, 4].

In Malaysia, the first case of COVID-19 was reported on January 25, 2020 [5]. The rapid escalation in COVID-19 infection rates has had a substantial impact on frontline healthcare providers both physically and emotionally. In addition to the increased workload due to the shortage of staff to care for COVID-19 patients, frontline healthcare providers have faced the possibility of being exposed to and infected with COVID-19 [6]. The mental stress has placed an undue burden on healthcare providers since they have to work in relatively confined spaces, wear thick isolation clothes, and care for large numbers of anxious patients [7].

Health professionals are often exposed to indirect trauma when working with traumatized patients. This puts them at risk of developing vicarious traumatization [8]. Vicarious traumatization refers to “the transformation in the inner experience of the therapist that comes about as a result of empathic engagement with client’s trauma material” [9]. It is also known as a secondary traumatic stress reaction [10]. It can result in disruptions in normal cognitive schemas with emerging symptoms of negative cognition, fluctuating moods, poor concentration, flashbacks, bad dreams, and intrusive thoughts or memories associated with the trauma of their patients [8].

Vicarious traumatization may be caused by several factors, including a high workload, a lack of protective equipment, an ineffective infection control system, and poor patient attitudes manifesting as, for example, verbal insults directed at medical staff [11]. The level of vicarious traumatization among healthcare providers during the COVID-19 pandemic in China was found to be lowest among frontline nurses compared to non-frontline nurses and the general public [12].

A study involving 1,060 participants (the majority of whom were healthcare providers) in China reported that more than 70% had moderate and high levels of psychological symptoms with elevated scores for obsessive compulsive behaviors, interpersonal sensitivity, phobic anxiety, and psychoticism [13]. One study reported that approximately one-third of healthcare providers had sustained a significant psychological impact [14].

A commonly used method of measuring vicarious traumatization is the Traumatic Stress Institute Life Events Checklist [15]. Another such instrument is the Traumatic Stress Institute Belief Scale–Revision L, which consists of an 80-item questionnaire that assesses levels of disruption among the five separate domains of safety, trust, control, esteem, and intimacy [16]. Vicarious traumatization can also be evaluated using the Secondary Traumatic Stress Scale. This instrument’s 17 items make it possible to verify the presence and relative frequency of symptoms [17].

In the fight against the COVID-19 pandemic, frontline healthcare providers have been facing enormous pressure stemming from a high risk of infection with inadequate protection from contamination and minimal support in coping with overwork, frustration, and exhaustion. These stressors impose significant psychological and mental health concerns. Protection from “a mental health injury” is essential for healthcare providers so that they can offer the best possible medical services to patients with COVID-19. It is, therefore, crucial that frontline healthcare providers are able to maintain a stable psychological and mental state when working during the COVID-19 crisis. Accordingly, early professional assessment and subsequent interventions are necessary.

Many studies have reported that frontline healthcare providers were more likely affected psychologically with depression, anxiety, stress, and insomnia than non-frontline healthcare providers [18, 19]. However, some studies have reported that non-frontline healthcare providers experienced more psychological impact than frontline healthcare providers during the COVID-19 pandemic [12, 20, 21]. This study aimed to compare the levels of vicarious traumatization between frontline and non-frontline healthcare providers in response to the COVID-19 pandemic.

## Materials and methods

### Population and sample

This comparative cross-sectional study was conducted among healthcare providers who were managing patients with suspected COVID-19 and those diagnosed with COVID-19 between May and July 2020 in two hospitals in Kelantan, Malaysia. Healthcare providers, including doctors, nurses, and medical assistants, aged less than 60 years old were recruited. Those diagnosed with any psychiatric illnesses were excluded. In this study, frontline healthcare providers refer to designated staffs in the healthcare facility that provide direct care to patients with confirmed or suspected COVID-19. Meanwhile, non-frontline healthcare providers refer to those dedicated to standard hospital functioning. Convenience sampling was applied, and frontline and non-frontline healthcare providers were identified and invited to participate in the study.

The sample size was calculated by comparing two means using the Power and Sample Size Calculation software version 3.0.43 (Microsoft Corporation, 2012). This study formed part of a larger study on the psychological impact of the COVID-19 pandemic. The variable that yielded the biggest sample size was anxiety. The standard deviation of the anxiety score among the non-frontline healthcare providers was 10.6 [22]. The detectable difference of 3.5 was decided after considering its clinical importance. After allowing for an alpha of 0.05, a power of 80%, and a non-response rate of 10%, the calculated sample size was 160 for non-frontline and 160 for frontline healthcare providers.

### Research tools

The case report form consisted of response for socioeconomic data, the Vicarious Traumatization Questionnaire, and the Medical Outcome Study Social Support Survey. In terms of sociodemographic data, responses were required in relation to age, race, marital status, number of children, education level, and household income as well as occupational information, including occupation, duration of employment, duration of work, shift work, and type of work.

Vicarious Traumatization Questionnaire has been used for the general public and members and non-members of medical teams aiding in COVID-19 [12]. It comprises a total of 38 items, which are composed of two dimensions, namely, physiological responses (11 items) and psychological responses (i.e., emotional responses [nine items], behavioral responses [seven items], cognitive responses [five items], and life belief [six items]) [12]. The scores for each question ranged from 0 (never) to 5 (always), and the total raw scores were used. Scores can range from 0 to 190, with higher scores indicating more vicarious traumatization. The Cronbach’s alpha for the questionnaire was 0.93, and the values for each dimension ranged from 0.73 to 0.92. The psychometric properties of the Malay-version Vicarious Traumatization Questionnaire were tested among 352 healthcare providers. The overall Cronbach’s alpha was 0.97 and best fit (Chi-squared/degree of freedom = 4.73; Tucker-Lewis index = 0.94; comparative fit index = 0.94; and root mean square error of approximation = 0.10) [23].

The Malay-version Medical Outcome Study (MOS) Social Support Survey consists of four dimensions, namely, emotional/informational (EMI) support, tangible (TAN) support, affectionate (AFF) support, and positive social interaction (POS), and comprises 16 items in total [24]. The EMI support dimension estimates the extent to which interpersonal relationships provide guidance, positive affect, and empathetic understanding (six items). TAN support pertains to the provision of material aid or behavioral assistance (three items), while AFF support measures expressions of love and affection (three items). Finally, POS relates to the availability of someone with whom to have fun (four items). Each item is rated on a five-point scale ranging from 1 (none of the time) to 5 (all of the time), with higher scores indicating more support. In this study, the raw scores for each dimension and the overall functional social support scores were transformed to a percent score. The composite reliability of the domains ranged from 0.649 to 0.903, the average variance of the domains ranged from 0.390 to 0.699, and the Cronbach’s alpha of the domains was 0.616–0.902.

### Data collection

Respondents were invited through an online method. Google Forms was used to develop the case report form, which was then distributed through the WhatsApp groups application at the administrative and departmental levels with gentle reminders. A virtual consent form that included the eligibility criteria for participation was distributed. They were informed that their participation was voluntary and that they could withdraw at any time. Those eligible and agreed to participate in the study were requested to respond and complete a self-administered questionnaire. The respondents were not required to sign in to a Google account to fill in the survey. The data collection continued until reaching the sample size. The respondents who were identified as being at risk of developing significant psychological conditions were referred for counselling and psychological support to the Psychological First Aid team.

### Data entry and analysis

The data were entered and analyzed using IBM SPSS Statistics version 26.0 (SPSS Inc., 2019). The data were checked and cleaned before the analysis was conducted. An independent *t*-test and analysis of covariance (ANCOVA) were performed. The dependent variable was the vicarious traumatization scores. The grouping variable was the frontline and non-frontline healthcare providers, and the controlled variables were sex, duration of employment, and social support.

### Ethics approval

Ethics approval was obtained from the Universiti Sains Malaysia Human Research Ethics Committee (USM/JEPeM/COVID19-10) and the Ministry of Health Medical Research Ethics Committee (NMRR-20-703-54576).

## Results

A total of 306 healthcare providers participated in this study: frontline group (n = 160) and non-frontline group (n = 146) with a response rate of 100.0% and 91.3%, respectively. The socioeconomic characteristics of the frontline and non-frontline healthcare providers are shown in Table 1.

**Table 1:** Socio-economic characteristics of healthcare providers.

	Frontline		Non-frontline	
Variables	n	(%)	n	(%)
Age (years)^a^	38.0	(6.21)	38.5	(7.13)
Household income (RM)^a^^,^^b^	5754.7	(3121.83)	4369.1	(2726.48)
Number of children^a^	2.5	(1.53)	2.3	(1.57)
Duration of employment (years)^a^	12.7	(5.84)	12.4	(7.01)
Sex				
Male	19	(11.9)	42	(28.8)
Female	141	(88.1)	104	(71.2)
Race				
Malay	156	(97.5)	145	(99.3)
Non-Malay	4	(2.5)	1	(0.7)
Education level				
Diploma	134	(83.8)	135	(92.4)
Bachelor	18	(11.2)	9	(6.2)
Master	8	(5.0)	2	(1.4)
Marital status				
Married	133	(83.1)	132	(90.4)
Unmarried	27	(16.9)	14	(9.6)
Occupation				
Paramedics	137	(85.6)	139	(95.2)
Medical staffs	23	(14.4)	7	(4.8)
Department				
Medical	54	(33.8)	24	(16.4)
Emergency	2	(1.3)	51	(34.9)
Intensive Care Unit	67	(41.9)	0	(0)
Surgical	14	(8.8)	56	(38.4)
Obstetric Gynaecology	5	(3.1)	0	(0)
Others	18	(11.2)	15	(10.2)
Shift work				
No	154	(96.2)	137	(93.8)
Yes	6	(3.8)	9	(6.2)
Social support				
Emotional support^a^	70.3	(25.44)	58.5	(27.93)
Tangible support^a^	64.4	(28.21)	56.2	(29.13)
Affectionate support^a^	74.7	(25.55)	62.8	(28.70)
Positive social interaction^a^	74.2	(24.48)	62.5	(27.51)

^a^ Expressed as mean (standard deviation)

^b^ Missing values (Frontline, n = 128; non-frontline, n = 87)

For the items relating to vicarious traumatization in healthcare providers, the mean score of the non-frontline group was higher than that of the frontline group for most of the scale items. For items 11, 14, 27, and 28, the differences in the mean scores were not statistically significant between the two groups (Table 2).

**Table 2:** Vicarious Traumatization Questionnaire items.

		Frontline (n = 160)		Non-frontline (n = 146)			
No.	Items	Mean	(SD)^a^	Mean	(SD)^a^	t (*df*^b^)	p value
1	In the face of choice, I have a hard time making a decision	2.4	(0.86)	2.5	(0.75)	-1.8 (304)	0.076
2	I feel hesitant when letting me do something alone	2.2	(0.83)	2.4	(0.84)	-2.7 (304)	0.008
3	I worry that I will say the wrong thing or do something wrong	2.4	(0.85)	2.5	(0.86)	-1.9 (304)	0.060
4	In work and life, I depend on others	1.9	(0.87)	2.3	(0.89)	-3.6 (304)	<0.005
5	I lose my temper with little things	1.9	(0.78)	2.2	(0.94)	-3.2 (282.18)	0.001
6	I deliberately avoided some topics and situations related to the disaster	1.7	(0.84)	2.1	(0.96)	-4.3 (289.78)	<0.005
7	I think human life is fragile	2.0	(1.11)	2.2	(1.07)	-1.5 (304)	0.128
8	I don’t feel others understand me	2.1	(1.01)	2.4	(0.96)	-2.2 (304)	0.026
9	I feel that telling someone what in my heart can cause trouble	2.4	(1.01)	2.5	(0.92)	-1.4 (304)	0.172
10	I feel overwhelmed in the face of unexpected things	2.6	(1.02)	2.7	(0.98)	-1.2 (304)	0.224
11	I feel my mood has been fluctuating recently	2.4	(1.22)	2.2	(0.99)	1.7 (300.08)	0.082
12	I dreamed of scary scenes related to disaster	1.7	(0.98)	2.0	(1.13)	-3.1 (288.17)	0.002
13	I lose interest in many things	1.8	(1.00)	2.1	(1.06)	-2.4 (304)	0.020
14	Thoughts related to disaster relief suddenly came to my mind	2.4	(1.20)	2.2	(0.98)	1.0 (300.16)	0.320
15	I don’t feel determined to do things	1.6	(0.87)	2.1	(1.07)	-4.2 (278.90)	< .005
16	I think this society is unfair	1.8	(1.15)	2.2	(1.09)	-2.7 (304)	0.007
17	I can’t rest assured that my goal is achieved as planned	1.9	(0.89)	2.3	(1.04)	-3.5 (286.96)	0.001
18	I feel frightened	1.9	(0.99)	2.1	(1.02)	-1.9 (304)	0.059
19	I can’t help thinking of disaster-related situations over and over again	1.9	(0.99)	2.1	(1.00)	-2.0 (300.69)	0.050
20	I feel bad when working or studying	1.9	(1.07)	2.1	(1.02)	-1.4 (304)	0.152
21	I feel depressed	2.1	(1.11)	2.2	(0.95)	-0.7 (304)	0.487
22	I don’t want to participate in group activities	1.8	(1.02)	2.2	(1.04)	-3.3 (304)	0.001
23	I seem to hear a call for help	1.3	(0.72)	1.9	(1.07)	-5.9 (249.95)	<0.005
24	I can’t feel the concern of people around me	1.8	(0.91)	2.1	(0.95)	-2.5 (304)	0.012
25	I feel I can’t do things lastingly	1.8	(0.98)	2.1	(1.02)	-2.4 (304)	0.019
26	Disaster-related scenes or sights suddenly invade my mind	1.8	(1.02)	2.1	(0.99)	-1.7 (304)	0.091
27	Faced with stress, I feel exhausted	2.6	(1.14)	2.5	(0.86)	1.2 (293.27)	0.228
28	Insomnia	2.2	(1.13)	2.2	(1.00)	0.2 (304)	0.873
29	Have a nightmare	1.7	(0.93)	2.0	(0.94)	-3.2 (304)	0.002
30	No appetite	1.6	(0.81)	2.0	(0.97)	-4.3 (282.79)	<0.005
31	Disgusting and vomiting	1.4	(0.79)	2.0	(1.13)	-5.7 (256.16)	<0.005
32	Gastrointestinal discomfort	1.6	(0.85)	2.2	(1.13)	-4.9 (268.65)	<0.005
33	Cramps, cramps	1.6	(0.91)	2.3	(1.23)	-5.1 (285.95)	<0.005
34	Backache	2.1	(1.09)	2.6	(1.29)	-3.5 (285.84)	0.001
35	Increased frequency of going to the toilet	1.7	(0.92)	2.3	(1.28)	-4.9 (261.95)	<0.005
36	Chest tightness, flustered	1.4	(0.77)	2.2	(1.32)	-5.8 (230.10)	<0.005
37	Headache, dizziness and bloating	1.9	(0.97)	2.6	(1.29)	-5.1 (268.57)	<0.005
38	Tired	2.5	(1.12)	2.9	(1.30)	-2.5 (304)	0.015

^a^ Standard deviation

^b^ Degree of freedom

The Vicarious Traumatization Questionnaire subscales are presented in Table 3. The highest mean scores for the Vicarious Traumatization Questionnaire subscales were in the area of psychological responses with a mean (standard deviation, SD) of 54.0 (17.71) for the frontline healthcare providers and 60.2 (20.13) for the non-frontline healthcare providers. The lowest mean scores for vicarious traumatization were for the subscale item cognitive response (frontline healthcare providers: mean [SD] = 9.0 [3.80], non-frontline healthcare providers: mean [SD] = 10.3 [4.40]).

**Table 3:** Vicarious Traumatization Questionnaire subscales.

	Frontliners (n = 160)		Non-frontliners (n = 146)				
Subscales	Mean	(SD)^a^	Mean	(SD)^a^	Mean diff^b^	t (*df*^c^)	p value
Physiological responses	19.7	(7.73)	25.2	(11.12)	-5.4	-4.9 (255.81)	<0.005
Psychological responses:	54.0	(17.71)	60.2	(20.13)	-6.3	-2.9 (290.21)	0.004
Behavioral responses	14.1	(4.19)	16.2	(4.91)	-2.1	-4.0 (286.27)	<0.005
Emotional responses	18.9	(6.87)	20.2	(6.86)	-1.3	-1.6 (304)	0.103
Cognitive responses	9.0	(3.80)	10.3	(4.40)	-1.3	-2.7 (287.77)	0.007
Life beliefs	11.89	(4.40)	13.49	(4.83)	-1.60	-3.02 (293.98)	0.003
Vicarious traumatization	73.69	(23.85)	85.40	(30.03)	-11.7	-3.79 (276.40)	<0.005

^a^ Standard deviation

^b^ mean difference

^c^ Degree of freedom

A comparison of the vicarious traumatization levels between the frontline and non-frontline healthcare providers is shown in Table 4. The vicarious traumatization overall mean score for the frontline healthcare providers was normally distributed and ranged from 41 to 164 with a mean (SD) of 73.7 (23.85). The vicarious traumatization overall mean score for the non-frontline healthcare providers was normally distributed and ranged from 38 to 148 with a mean (SD) of 85.4 (30.03). The independent *t*-test analysis showed a significant difference in the vicarious traumatization scores between the frontline and non-frontline healthcare providers (p < 0.005) with a mean difference of −11.7 in the scores. ANCOVA showed a significant difference in the estimated marginal mean of the vicarious traumatization scores between the frontline and non-frontline healthcare providers with a mean difference of −5.4 after adjusting for sex, duration of employment, and social support (p = 0.031).

**Table 4:** Comparison of vicarious traumatization levels between frontline and non-frontline healthcare providers.

Groups	n	Desc. Mean^a^	(SD)^b^	EMM^c^ (95%CI^d^)	Mean diff^e^ (95%CI^d^)	F stat^f^ (*df*^g^)	p value ^h^
Frontline	160	73.7	(23.85)	74.3 (68.26; 80.37)	-5.4 (-13.01; 2.21)	4.7 (1)	0.031
Non-Frontline	146	85.4	(30.03)	79.7 (75.12; 84.30)			

^a^ Descriptive mean

^b^ Standard deviation

^c^ Estimated marginal mean

^d^ Confidence interval

^e^ Mean difference

^f^ *F* statistic

^g^ Degree of freedom

^h^ Analysis of covariance. Adjusted for gender, duration of employment and social support

There were two significant interactions: between the groups and sex, and the groups and the social support scores. The subgroups analysis showed that the female group and a high score for social support were significantly different in the vicarious traumatization mean score between the frontline and non-frontline groups. The interactions were included in the model. All the model assumptions were met. The residual plots indicated that the overall model fitness assumption was satisfied. The normality of the standardized residuals was appropriate. The variable functional form for the MOS social support score was appropriate. There were no obvious outliers when plotting the standardized residuals against the predicted value. The ANCOVA analysis confirmed the higher vicarious traumatization of non-frontline healthcare providers by 5.4 scores compared to the frontline healthcare providers after adjusting for sex, duration of employment, and social support.

## Discussion

This comparative cross-sectional study showed that the level of vicarious traumatization was higher in the non-frontline healthcare providers compared to those on the frontline during the COVID-19 pandemic after adjusting for sex, duration of employment, and social support. This result was similar to that of a study in China [12] and further provides support for a previous study that found that physicians and nurses who work in COVID-19 wards had a lower frequency of burnout compared to medical staff in non-COVID wards [25]. Burnout is one of the factors that contributes to vicarious traumatization. Many other factors may influence the severity of vicarious traumatization, including gender, marital status, an extension of the working period, less awareness of the pandemic, long-term high pressure, and stressful conditions when fighting COVID-19 [12].

Frontline healthcare providers have more exposure and are well-prepared physically and psychologically to battle this pandemic. They would have learned to manage their physical, psychology, and mental health prior to the COVID-19 pandemic as their working environment requires them to face vicarious traumatization due to their workloads and work-related stress [26]. Prior knowledge and/or previous experience handling situations like the severe acute respiratory syndrome or Middle East respiratory syndrome outbreaks, which were similar but less severe than the current pandemic, are factors that may contribute to the lower severity of vicarious traumatization among frontline healthcare providers [12]. A study reported that the majority of non-frontline respondents were less experienced, irrespective of whether they had been newly recruited or had moved from their specialist area since many skilled and experienced clinicians had been deployed to frontline positions [27]. The current study reported a slightly shorter duration of employment among the non-frontline healthcare providers. The higher score for the non-frontline healthcare providers showed that this group suffered more psychologically compared to the frontline group. Non-frontline healthcare providers may have less psychological endurance [12]. Having less access to formal psychological support, less firsthand medical information on the outbreak, and less intensive training on personal protective equipment and infection control measures [20] also may have contributed to the increased severity of vicarious traumatization among the healthcare providers in the non-frontline group.

Working in departments that are not prepared for COVID-19 cases may affect the psychological responses of healthcare providers since they may have an increased risk of exposure to COVID-19 infection, especially if patients are asymptomatic [28]. Full personnel protective equipment is a must to protect staff from the transmission of the infection. However, the shortage of such equipment during the pandemic may have influenced their psychological and work performance [7, 29]. This equipment has to be prioritized for distribution to the COVID-19 isolation wards, and non-COVID ward staff must continue to do their work as usual. Non-frontline healthcare providers are therefore more likely to be exposed to nosocomial infections [30]. Non-emergency cases or surgeries have consequently had to be postponed to reduce the risk of exposure to nosocomial infections since the viruses can spread easily from one person to another.

In the present study, the severity of vicarious traumatization was highest in the psychological responses dimension in both groups. The psychological responses consisted of the subscales of behavioral responses, emotional responses, cognitive responses, and life beliefs. The emotional responses subscale produced the highest score among the subscales. This result parallels that of a study in China [12]. During this pandemic, most healthcare providers have experienced feelings of stress, depression, anxiety, fatigue, and burnout since their workloads are greater than usual. Heavy workload has been found to be one of the COVID-19 risk factors [31]. Some healthcare providers have decided to quarantine themselves from their family, colleagues, and the public to prevent transmission of the virus. During the quarantine period, they may feel sympathy for patients with COVID-19 and concerned for those who work as healthcare providers [12], which can trigger psychological responses. In addition, stigmatization from the public as “carrier[s] of the virus” since they are working at hospitals or health centers can have an impact on their psychological state [32].

The internet and other media have a role to play in disseminating information about COVID-19. Many people use these facilities as information sources. Sometimes, the information is wholly or partially inaccurate, which can contribute to people’s psychological responses and affect the level of vicarious traumatization, particularly among healthcare providers or the general public who have no medical background, less knowledge about the pandemic, or weak psychological endurance [12]. However, the government, together with the involved authorities, have set up strategies to deliver accurate information about COVID-19 and prevent the spread of false information. On the other hand, psychological support can be provided through the media, which can provide less personalized sources of care information via, for example, publication-style psychological materials and psychological resources [33]. Use of online mental health self-assessments such as the Depression, Anxiety, and Stress Scale-21 to determine the severity of one’s mental health status before seeking professional counseling or psychotherapy is recommended [34]. Helpline support should also be available for those who need to talk to someone as these services are provided by qualified mental health first aiders [32].

Our study has some limitations. First is the inherent nature of the sample, including sampling method being only applicable to people with internet access and recall bias, are possibilities inherent in the survey study. The limited sample size and less representative subjects for analysis make it difficult to generalise the findings. In the study, despite the limitations, the potential confounding factor, particularly concerning gender differences, was adjusted to the extent possible.

## Conclusion

All healthcare providers face vicarious traumatization, but particularly so during this COVID-19 pandemic. The level of vicarious traumatization was higher among non-frontline compared to frontline healthcare providers. However, the level of severity may differ from person to person, depending on how they handle their physical, psychological, and mental health. Hence, support from various resources, such as colleagues, family, the general public, and the government, may play an essential role in the mental health of healthcare providers.
